# Current Awareness on Comparative and Functional Genomics

**DOI:** 10.1002/cfg.352

**Published:** 2004-04


**Comparative and Functional Genomics**
*Comp Funct Genom* 2004; **5**: 295-302
Published online in Wiley InterScience (www.interscience.wiley.com). **DOI:** 10.1002/cfg.352

### Current awareness on comparative and functional genomics

CURRENT AWARENESS - USE THE LINKS ONLINE

View this bibliography online at WILEY InterScience www.interscience.wiley.com

Your gateway to Wiley journals online

The articles use crossref, the innovative multi-publisher reference linking. This enables readers to move seamlessly from the reference to the cited publication, typically located on a different server and published by a different publisher. Links to MEDLINE abstracts are also available for key journals in the biosciences.

2/45

### 1 Reviews & symposia

Aloy P, Russell RB. 2003. Understanding and predicting protein assemblies with 3D structures (Conference review). *Comp Funct Genom* **4:** (4) 410.
Armstrong SA, Gohub TR, Korsmeyer SJ. 2003. *MLL*-rearranged leukemias: Insights from gene expression profiling. *Semin Hematol* **40:** (4) 268.
Arranz MJ, Kerwin RW. 2003. Pharmacogenetic and pharmacogenomic research for the prediction of response to antipsychotics in schizophrenia. *Drug Dev Res* **60:** (2) 104.
Basik M, Mousses S, Trent J. 2003. Integration of genomic technologies for accelerated cancer drug development. *Biotechniques* **35:** (3) 580.
Bauer A, Beckmann B, Busold C, Brandt O, Kusnezow W, Pullat J, Aign V, Fellenberg K, Fleischer R, Jacob A, Frohme M, Hoheisel JD. 2003. Use of complex DNA and antibody microarrays as tools in functional analyses (Conference review). *Comp Funct Genom* **4:** (5) 520.
Bell RE, Ben-Tal N. 2003. *In silico* identification of functional protein interfaces (Conference review). *Comp Funct Genom* **4:** (4) 420.
Davids W, Fuxelius HH, Andersson SGE. 2003. The journey to smORFland (Conference review). *Comp Funct Genom* **4:** (5) 537.
Davidsson P, Brinkmalm A, Karlsson G, Persson R, Lindbjer M, Puchades M, Folkesson S, Paulson L, Dahl A, Rymo L, Silberring J, Ekman R, Blennow K. 2003. Clinical mass spectrometry in neuroscience. Proteomics and peptidomics. *Cell Mol Biol (Noisy-le-Grand)* **49:** (5) 681.
De Bono B, Trowsdale J. 2003. Exploring the immunogenome with bioinformatics. *Semin Immunol* **15:** (4) 233.
DeFilippis V, Raggo C, Moses A, Fruh K. 2003. Functional genomics in virology and antiviral drug discovery (Review). *Trends Biotechnol* **21:** (10) 452.
Desai AA, Innocenti F, Ratain MJ. 2003. Pharmacogenomics: Road to anticancer therapeutics nirvana? *Oncogene* **22:** (42) 6621.
Dowsey AW, Dunn MJ, Yang GZ. 2003. The role of bioinformatics in two-dimensional gel electrophoresis. *Proteomics* **3:** (8) 1567.
Feldker DEM, De Kloet ER, Kruk MR, Datson NA. 2003. Large-scale gene expression profiling of discrete brain regions: Potential, limitations, and application in genetics of aggressive behavior. *Behav Genet* **33:** (5) 537.
Ferguson PL, Smith RD. 2003. Proteome analysis by mass spectrometry. *Annu Rev Biophys Biomol Struct* **32:** 399.
Ferrando AA, Look AT. 2003. Gene expression profiling in T-cell acute lymphoblastic leukemia. *Semin Hematol* **40:** (4) 274.
Ferre F, Via A, Ausiello G, Brannetti B, Zanzoni A, Helmer-Citterich M. 2003. Development of computational tools for the inference of protein interaction specificity rules and functional annotation using structural information (Conference review). *Comp Funct Genom* **4:** (4) 416.
Frolov AE, Godwin AK, Favorova OO. 2003. Differential gene expression analysis by DNA microarray technology and its application in molecular oncology (Review). *Mol Biol-Engl Tr* **37:** (4) 486.
Gammill LS, Bronner-Fraser M. 2003. Neural crest specification: Migrating into genomics. *Nat Rev Neurosci* **4:** (10) 795.
Goldsmith-Fischman S, Honig B. 2003. Structural genomics: Computational methods for structure analysis (Review). *Protein Sci* **12:** (9) 1813.
Haferlach T, Kohlmann A, Kern W, Hiddemann W, Schnittger S, Schoch C. 2003. Gene expression profiling as a tool for the diagnosis of acute leukemias. *Semin Hematol* **40:** (4) 281.
Hellerstein MK. 2003. *In vivo* measurement of fluxes through metabolic pathways: The missing link in functional genomics and pharmaceutical research. *Annu Rev Nutr* **23:** 379.
Henikoff S, Comai L. 2003. Single-nucleotide mutations for plant functional genomics. *Annu Rev Plant Biol* **54:** 375.
Hilson P, Small I, Kuiper MTR. 2003. European consortia building integrated resources for *Arabidopsis* functional genomics. *Curr Opin Plant Biol* **6:** (5) 426.
Ito C, Ouchi Y. 2003. Toward schizophrenia genes: Genetics and transcriptome. *Drug Dev Res* **60:** (2) 111.
Johnsson N, Johnsson K. 2003. A fusion of disciplines: Chemical approaches to exploit fusion proteins for functional genomics (Review). *Chembiochem* **4:** (9) 803.
Jones S, Thornton JM. 2003. Protein-DNA interactions: The story so far and a new method for prediction (Conference review). *Comp Funct Genom* **4:** (4) 428.
Kiechle FL, Holland-Staley CA. 2003. Genomics, transcriptomics, proteomics, and numbers. *Arch Pathol Lab Med* **127:** (9) 1089.
Klink R, Boksa P, Joober R. 2003. Pharmacogenomics and animal models of schizophrenia. *Drug Dev Res* **60:** (2) 95.
Koj A, Jura J. 2003. Complex analysis of genes involved in the inflammatory response: Interleukin-1 induced differential transcriptome of cultured human hepatoma HepG2 cells (Review). *Acta Biochim Pol* **50:** (3) 573.

In order to keep subscribers up-to-date with the latest developments in their field, this current awareness service is provided by John Wiley & Sons and contains newly-published material on comparative and functional genomics. Each bibliography is divided into 16 sections. 1 Reviews & symposia; 2 General; 3 Large-scale sequencing and mapping; 4 Evolutionary genomics; 5 Comparative genomics; 6 Pathways, gene families and regulons; 7 Pharmacogenomics; 8 EST, cDNA and other clone resources; 9 Functional genomics; 10 Transcriptomics; 11 Proteomics; 12 Protein structural genomics; 13 Metabolomics; 14 Genomic approaches to development; 15 Technological advances; 16 Bioinformatics. Within each section, articles are listed in alphabetical order with respect to author. If, in the preceding period, no publications are located relevant to any one of these headings, that section will be omitted.

Copyright © 2004 John Wiley & Sons, Ltd.

Landegren U, Dahl F, Nilsson M, Fredriksson S, Baner J, Gullberg M, Jarvius J, Gustafsdottir S, Soderberg O, Ericsson O, Stenberg J, Schallmeier E. 2003. Padlock and proximity probes for *in situ* and array-based analyses: Tools for the post-genomic era (Conference review). *Comp Funct Genom* **4:** (5) 525.

Lefkovits I. 2003. Quantitative proteomics of lymphocytes (Conference review). *Comp Funct Genom* **4:** (5) 531.

Linial M. 2003. Fishing with (Proto)Net: A principled approach to protein target selection (Conference review). *Comp Funct Genom* **4:** (5) 542.

Martelli PL, Fariselli P, Tasco G, Casadio R. 2003. The prediction of membrane protein structure and genome structural annotation (Conference review). *Comp Funct Genom* **4:** (4) 406.

Pallen MJ, Chaudhuri RR, Henderson IR. 2003. Genomic analysis of secretion systems. *Curr Opin Microbiol* **6:** (5) 519.

Pawliczak R, Shelhamer JH. 2003. Application of functional genomics in allergy and clinical immunology. *Allergy* **58:** (10) 973.

Ping PP. 2003. Identification of novel signaling complexes by functional proteomics (Review). *Circ Res* **93:** (7) 595.

Russo G, Zegar C, Giordano A. 2003. Advantages and limitations of microarray technology in human cancer. *Oncogene* **22:** (42) 6497.

Saviozzi S, Calogero RA. 2003. Microarray probe expression measures, data normalization and statistical validation (Conference review). *Comp Funct Genom* **4:** (4) 442.

Scheidegger KA, Payne GA. 2003. Unlocking the secrets behind secondary metabolism: A review of *Aspergillus flavus* from pathogenicity to functional genomics. *J Toxicol-Toxin Rev* **22:** (2-3) 423.

Schuler MA, Werck-Reichhart D. 2003. Functional genomics of P450s. *Annu Rev Plant Biol* **54:** 629.

Siew N, Fischer D. 2003. Unravelling the ORFan puzzle (Conference review). *Comp Funct Genom* **4:** (4) 432.

Stone LS, Vulchanova L. 2003. The pain of antisense: *In vivo* application of antisense oligonucleotides for functional genomics in pain and analgesia. *Advan Drug Delivery Rev* **55:** (8) 1081.

Tramontano A. 2003. Comparative modelling techniques: Where are we? (Conference review). *Comp Funct Genom* **4:** (4) 402.

Valencia A. 2003. Multiple sequence alignments as tools for protein structure and function prediction (Conference review). *Comp Funct Genom* **4:** (4) 424.

Weckwerth W. 2003. Metabolomics in systems biology. *Annu Rev Plant Biol* **54:** 669.

Yuan X, Shao Y, Bystroff C. 2003. *Ab initio* protein structure prediction using pathway models (Conference review). *Comp Funct Genom* **4:** (4) 397.

Zhan FH, Barlogie B, Shaughnessy J. 2003. Toward the identification of distinct molecular and clinical entities of multiple myeloma using global gene expression profiling. *Semin Hematol* **40:** (4) 308.

Zhu H, Bilgin M, Snyder M. 2003. Proteomics. *Annu Rev Biochem* **72:** 783.

Zhu T. 2003. Global analysis of gene expression using GeneChip microarrays. *Curr Opin Plant Biol* **6:** (5) 418.

Zierhut M, Mizuki N, Ohno S, Inoko H, Gul A, Onoe K, Isogai E. 2003. Immunology and functional genomics of Behcet's disease. (Review) *Cell Mol Life Sci* **60:** (9) 1903.

### 3 Large-scale sequencing and mapping

Baar C, Eppinger M, Raddatz G, Simon J, Lanz C, Klimmek O, Nandakumar R, Gross R, Rosinus A, Keller H *et al*. 2003. Complete genome sequence and analysis of *Wolinella succinogenes*. *Proc Natl Acad Sci U S A* **100:** (20) 11690.

Buell CR, Joardar V, Lindeberg M, Selengut J, Paulsen IT, Gwinn ML, Dodson RJ, Deboy RT, Durkin AS, Kolonay JF *et al*. 2003. The complete genome sequence of the *Arabidopsis* and tomato pathogen *Pseudomonas syringae* pv. *tomato* DC3000. *Proc Natl Acad Sci U S A* **100:** (18) 10181.

De Almeida DF, Hungria M, Guimaraes CT, Antonio RV, Almeida FC, De Almeida LGP, De Almeida R, Alves-Gomes JA, Andrade EM, Araripe J *et al*. 2003. The complete genome sequence of *Chromobacterium violaceum* reveals remarkable and exploitable bacterial adaptability. *Proc Natl Acad Sci U S A* **100:** (20) 11660.

Haas BJ, Delcher AL, Mount SM, Wortman JR, Smith RK, Hannick LI, Maiti R, Ronning CM, Rusch DB, Town CD *et al*. 2003. Improving the *Arabidopsis* genome annotation using maximal transcript alignment assemblies. *Nucleic Acids Res* **31:** (19) 5654.

Kalinowski J, Bathe B, Bartels D, Bischoff N, Bott M, Burkovski A, Dusch N, Eggeling L, Eikmanns BJ, Gaigalat L *et al*. 2003. The complete *Corynebacterium glutamicum* ATCC 13032 genome sequence and its impact on the production of L-aspartate-derived amino acids and vitamins. *J Biotechnol* **104:** (1-3) 5.

Kirkness EF, Bafna V, Halpern AL, Levy S, Remington K, Rusch DB, Delcher AL, Pop M, Wang W, Fraser CM *et al*. 2003. The dog genome: Survey sequencing and comparative analysis. *Science* **301:** (5641) 1898.

Nelson KE, Fleischmann RD, DeBoy RT, Paulsen IT, Fouts DE, Eisen JA, Daugherty SC, Dodson RJ, Durkin AS, Gwinn M *et al*. 2003. Complete genome sequence of the oral pathogenic bacterium *Porphyromonas gingivalis* strain W83. *J Bacteriol* **185:** (18) 5591.

Papazisi L, Gorton TS, Kutish G, Markham PF, Browning GF, Nguyen DK, Swartzell S, Madan A, Mahairas G, Geary SJ. 2003. The complete genome sequence of the avian pathogen *Mycoplasma gallisepticum* strain R-low. *Microbiology* **149:** (9) 2307.

Wortman JR, Haas BJ, Hannick LI, Smith RK, Maiti R, Ronning CM, Chan AP, Yu CH, Ayele M, Whitelaw CA *et al*. 2003. Annotation of the *Arabidopsis* genome. *Plant Physiol* **132:** (2) 461.

### 4 Evolutionary genomics

Achaz G, Coissac E, Netter P, Rocha EPC. 2003. Associations between inverted repeats and the structural evolution of bacterial genomes. *Genetics* **164:** (4) 1279.

Hinchliffe SJ, Isherwood KE, Stabler RA, Prentice MB, Rakin A, Nichols RA, Oyston PCF, Hinds J, Titball RW, Wren BW. 2003. Application of DNA microarrays to study the evolutionary genomics of *Yersinia pestis* and *Yersinia pseudotuberculosis*. *Genome Res* **13:** (9) 2018.

### 5 Comparative genomics

Golubov A, Heesemann J, Rakin A. 2003. Uncovering genomic differences in human pathogenic *Yersinia enterocolitica*. *FEMS Immunol Med Microbiol* **38:** (2) 107.

Momynaliev KT, Smirnova OV, Kudryavtseva LV, Govorun VM. 2003. Comparative genome analysis of *Helicobacter pylori* strains. *Mol Biol (Mosk)* **37:** (4) 529.

Parkhill J, Sebaihia M, Preston A, Murphy LD, Thomson N, Harris DE, Holden MTG, Churcher CM, Bentley SD, Mungall KL *et al*. 2003. Comparative analysis of the genome sequences of *Bordetella pertussis*, *Bordetella parapertussis* and *Bordetella bronchiseptica*. *Nat Genet* **35:** (1) 32.

Tauch A, Puhler A, Kalinowski J, Thierbach G. 2003. Plasmids in *Corynebacterium glutamicum* and their molecular classification by comparative genomics. *J Biotechnol* **104:** (1-3) 27.

### 6 Pathways, gene families and regulons

Breton G, Danyluk J, Charron JBF, Sarhan F. 2003. Expression profiling and bioinformatic analyses of a novel stress-regulated multispanning transmembrane protein family from cereals and *Arabidopsis*. *Plant Physiol* **132:** (1) 64.

Iourgenko V, Zhang WJ, Mickanin C, Daly I, Jiang C, Hexham JM, Orth AP, Miraglia L, Meltzer J, Garza D *et al*. 2003. Identification of a family of cAMP response element-binding protein coactivators by genome-scale functional analysis in mammalian cells. *Proc Natl Acad Sci U S A* **100:** (21) 12147.

Maathuis FJM, Filatov V, Herzyk P, Krijger GC, Axelsen KB, Chen SX, Green BJ, Li Y, Madagan KL, Sanchez-Fernandez R *et al*. 2003. Transcriptome analysis of root transporters reveals participation of multiple gene families in the response to cation stress. *Plant J* **35:** (6) 675.

McHardy AC, Tauch A, Ruckert C, Puhler A, Kalinowski J. 2003. Genome-based analysis of biosynthetic aminotransferase genes of *Corynebacterium glutamicum*. *J Biotechnol* **104:** (1-3) 229.

Copyright © 2004 John Wiley & Sons, Ltd. 

### 7 Pharmacogenomics

Absi TS, Sundt TM, Tung WS, Moon M, Lee JK, Damiano RR, Thompson RW. 2003. Altered patterns of gene expression distinguishing ascending aortic aneurysms from abdominal aortic aneurysms: Complementary DNA expression profiling in the molecular characterization of aortic disease. *J Thorac Cardiovasc Surg* **126:** (2) 344.

Agarwal AK, Rogers PD, Baerson SR, Jacob MR, Barker KS, Cleary JD, Walker LA, Nagle DG, Clark AM. 2003. Genome-wide expression profiling of the response to polyene, pyrimidine, azole, and echinocandin antifungal agents in *Saccharomyces cerevisiae*. *J Biol Chem* **278:** (37) 34998.

Baggerly KA, Morris JS, Wang J, Gold D, Xiao LC, Coombes KR. 2003. A comprehensive approach to the analysis of matrix-assisted laser desorption/ionization-time of flight proteomics spectra from serum samples. *Proteomics* **3:** (9) 1667.

Butterfield DA, Castegna A. 2003. Proteomic analysis of oxidatively modified proteins in Alzheimer's disease brain: Insights into neurodegeneration. *Cell Mol Biol (Noisy-le-Grand)* **49:** (5) 747.

Carrette O, Demalte I, Scherl A, Yalkinoglu O, Corthals G, Burkhard P, Hochstrasser DF, Sanchez JC. 2003. A panel of cerebrospinal fluid potential biomarkers for the diagnosis of Alzheimer's disease. *Proteomics* **3:** (8) 1486.

Chen LM, Goryachev A, Sun J, Kim P, Zhang H, Phillips MJ, MacGregor P, Lebel S, Edwards AM, Cao QF *et al*. 2003. Altered expression of genes involved in hepatic morphogenesis and fibrogenesis are identified by cDNA microarray analysis in biliary atresia. *Hepatology* **38:** (3) 567.

Chen W, Fu XB, Sun XQ, Sun TZ, Zhao ZL, Sheng ZY. 2003. Analysis of differentially expressed genes in keloids and normal skin with cDNA microarray. *J Surg Res* **113:** (2) 208.

Chen WNU, Yu LR, Strittmatter EF, Thrall BD, Camp DG, Smith RD. 2003. Detection of *in situ* labeled cell surface proteins by mass spectrometry: Application to the membrane subproteome of human mammary epithelial cells. *Proteomics* **3:** (8) 1647.

Chi JT, Chang HY, Haraldsen G, Jahnsen FL, Troyanskaya OG, Chang DS, Wang Z, Rockson SG, Van de Rijn M, Botstein D *et al*. 2003. Endothelial cell diversity revealed by global expression profiling. *Proc Natl Acad Sci U S A* **100:** (19) 10623.

Chowers I, Gunatilaka TL, Farkas RH, Qian J, Hackam AS, Duh E, Kageyama M, Wang CW, Vora A, Campochiaro PA *et al*. 2003. Identification of novel genes preferentially expressed in the retina using a custom human retina cDNA microarray. *Invest Ophthalmol Vis Sci* **44:** (9) 3732.

Clarke W, Silverman BC, Zhang Z, Chan DW, Klein AS, Molmenti EP. 2003. Characterization of renal allograft rejection by urinary proteomic analysis. *Ann Surg* **237:** (5) 660.

Coombes KR, Fritsche HA, Clarke C, Chen JN, Baggerly KA, Morris JS, Xiao LC, Hung MC, Kuerer HM. 2003. Quality control and peak finding for proteomics data collected from nipple aspirate fluid by surface-enhanced laser desorption and ionization. *Clin Chem* **49:** (10) 1615.

Copland JA, Luxon BA, Ajani L, Maity T, Campagnaro E, Guo HP, LeGrand SN, Tamboli P, Wood CG. 2003. Genomic profiling identifies alterations in TGFβ signaling through loss of TGFβ receptor expression in human renal cell carcinogenesis and progression. *Oncogene* **22:** (39) 6109.

De Longueville F, Atienzar FA, Marcq L, Dufrane S, Evrard S, Wouters L, Leroux F, Bertholet V, Gerin B, Whomsley R *et al*. 2003. Use of a low-density microarray for studying gene expression patterns induced by hepatotoxicants on primary cultures of rat hepatocytes. *Toxicol Sci* **75:** (2) 378.

Desiderio DM, Zhan X. 2003. The human pituitary proteome: The characterization of differentially expressed proteins in an adenoma compared to a control. *Cell Mol Biol (Noisy-le-Grand)* **49:** (5) 689.

Du JJ, Dou KF, Peng SY, Xiao HS, Wang WZ, Guan WX, Wang ZH, Gao ZQ, Liu YB. 2003. cDNA suppression subtraction library for screening down-regulated genes in gastric carcinoma. *World J Gastroenterol* **9:** (7) 1439.

Fachado A, Rodriguez A, Molina J, Silverio JC, Marino APMP, Pinto LMO, Angel SO, Infante JF, Traub-Cseko Y, Amendoeira RR *et al*. 2003. Long-term protective immune response elicited by vaccination with an expression genomic library of *Toxoplasma gondii*. *Infect Immun* **71:** (9) 5407.

Gadal F, Bozic C, Pillot-Brochet C, Malinge S, Wagner S, Le Cam A, Buffat L, Crepin M, Iris F. 2003. Integrated transcriptome analysis of the cellular mechanisms associated with Ha-ras-dependent malignant transformation of the human breast epithelial MCF7 cell line. *Nucleic Acids Res* **31:** (19) 5789.

Gadgil HS, Pabst KM, Giorgianni F, Umstot ES, Desiderio DM, Beranova-Giorgianni S, Gerling IC, Pabst MJ. 2003. Proteome of monocytes primed with lipopolysaccharide: Analysis of the abundant proteins. *Proteomics* **3:** (9) 1767.

Galindo CL, Sha J, Ribardo DA, Fadl AA, Pillai L, Chopra AK. 2003. Identification of *Aeromonas hydrophila* cytotoxic enterotoxin-induced genes in macrophages using microarrays. *J Biol Chem* **278:** (41) 40198.

Gonzalez-Maeso J, Yuen T, Ebersole BJ, Wurmbach E, Lira A, Zhou MM, Weisstaub N, Hen R, Gingrich JA, Sealfon SC. 2003. Transcriptome fingerprints distinguish hallucinogenic and nonhallucinogenic 5-hydroxytryptamine 2A receptor agonist effects in mouse somatosensory cortex. *J Neurosci* **23:** (26) 8836.

Grzeskowiak R, Witt H, Drungowski M, Thermann R, Hennig S, Perrot A, Osterziel KJ, Klingbiel D, Scheid S, Spang R *et al*. 2003. Expression profiling of human idiopathic dilated cardiomyopathy. *Cardiovasc Res* **59:** (2) 400.

Guillaume E, Zimmermann C, Burkhard PR, Hochstrasser DF, Sanchez JC. 2003. A potential cerebrospinal fluid and plasmatic marker for the diagnosis of Creutzfeldt-Jakob disease. *Proteomics* **3:** (8) 1495.

Hashimoto SI, Morohoshi K, Suzuki T, Matsushima K. 2003. Lipopolysaccharide-inducible gene expression profile in human monocytes. *Scand J Infec Dis* **35:** (9) 619.

Hayano T, Yanagida M, Yamauchi Y, Shinkawa T, Isobe T, Takahashi N. 2003. Proteomic analysis of human Nop56p-associated pre-ribosomal ribonucleoprotein complexes: Possible link between Nop56p and the nucleolar protein treacle responsible for Treacher Collins syndrome. *J Biol Chem* **278:** (36) 34309.

Hedvat CV, Comenzo RL, Teruya-Feldstein J, Olshen AB, Ely SA, Osman K, Zhang YN, Kalakonda N, Nimer SD. 2003. Insights into extramedullary tumour cell growth revealed by expression profiling of human plasmacytomas and multiple myeloma. *Br J Haematol* **122:** (5) 728.

Herrmann PC, Gillespie JW, Charboneau L, Bichsel VE, Paweletz CP, Calvert VS, Kohn EC, Emmert-Buck MR, Liotta LA, Petricoin EF. 2003. Mitochondrial proteome: Altered cytochrome *c* oxidase subunit levels in prostate cancer. *Proteomics* **3:** (9) 1801.

Hilario M, Kalousis A, Muller M, Pellegrini C. 2003. Machine learning approaches to lung cancer prediction from mass spectra. *Proteomics* **3:** (9) 1716.

Hiratsuka M, Inoue T, Toda T, Kimura N, Shirayoshi Y, Kamitani H, Watanabe T, Ohama E, Tahimic CGT, Kurimasa A *et al*. 2003. Proteomics-based identification of differentially expressed genes in human gliomas: Down-regulation of SIRT2 gene. *Biochem Biophys Res Commun* **309:** (3) 558.

Howard BA, Wang MZ, Campa MJ, Corro C, Fitzgerald MC, Patz EF. 2003. Identification and validation of a potential lung cancer serum biomarker detected by matrix-assisted laser desorption/ionization-time of flight spectra analysis. *Proteomics* **3:** (9) 1720.

Jenner RG, Maillard K, Cattini N, Weiss RA, Boshoff C, Wooster R, Kellam P. 2003. Kaposi's sarcoma-associated herpesvirus-infected primary effusion lymphoma has a gene expression profile. *Proc Natl Acad Sci U S A* **100:** (18) 10399.

Jin JY, Almon RR, Dubois DC, Jusko WJ. 2003. Modeling of corticosteroid pharmacogenomics in rat liver using gene microarrays. *J Pharmacol Exp Ther* **307:** (1) 93.

Kondo T, Seike M, Mori Y, Fujii K, Yamada T, Hirohashi S. 2003. Application of sensitive fluorescent dyes in linkage of laser microdissection and two-dimensional gel electrophoresis as a cancer proteomic study tool. *Proteomics* **3:** (9) 1758.

Kyng KJ, May A, Kolvraa S, Bohr VA. 2003. Gene expression profiling in Werner syndrome closely resembles that of normal aging. *Proc Natl Acad Sci U S A* **100:** (21) 12259.

Le Roch KG, Zhou YY, Blair PL, Grainger M, Moch JK, Haynes JD, De la Vega P, Holder AA, Batalov S, Carucci DJ *et al*. 2003. Discovery of gene function by expression profiling of the malaria parasite life cycle. *Science* **301:** (5639) 1503.

Lee M, Kwon J, Kim SN, Kim JE, Koh WS, Kim EJ, Chung MK, Han

Copyright © 2004 John Wiley & Sons, Ltd. 

SS, Song CW. 2003. cDNA microarray gene expression profiling of hydroxyurea, paclitaxel, and *p*-anisidine, genotoxic compounds with differing tumorigenicity results. *Environ Mol Mutagen* **42:** (2) 91.
Li C, Chen ZC, Xiao ZQ, Wu XY, Zhan XQ, Zhang XP, Li MY, Li JL, Feng XP, Liang SP *et al*. 2003. Comparative proteomics analysis of human lung squamous carcinoma. *Biochem Biophys Res Commun* **309:** (1) 253.
Lin Y, Huang RC, Chen LP, Lisoukov H, Lu ZH, Li SY, Wang CC, Huang RP. 2003. Profiling of cytokine expression by biotin-labeled-based protein-arrays. *Proteomics* **3:** (9) 1750.
Liu LX, Jiang HC, Liu ZH, Zhu AL, Zhou J, Zhang WH, Wang XQ, Wu M. 2003. Gene expression profiles of hepatoma cell line BEL-7402. *Hepatogastroenterology* **50:** (53) 1496.
Markey MK, Tourassi GD, Floyd CE. 2003. Decision tree classification of proteins identified by mass spectrometry of blood serum samples from people with and without lung cancer. *Proteomics* **3:** (9) 1678.
Mayers C, Duffield M, Rowe S, Miller J, Lingard B, Hayward S, Titball RW. 2003. Analysis of known bacterial protein vaccine antigens reveals biased physical properties and amino acid comparison. *Comp Funct Genom* **4:** (5) 468.
Mian S, Ball G, Hornbuckle J, Holding F, Carmichael J, Ellis I, Ali S, Li G, McArdle S, Creaser C, Rees R. 2003. A prototype methodology combining surface-enhanced laser desorption/ionization protein chip technology and artificial neural network algorithms to predict the chemoresponsiveness of breast cancer cell lines exposed to paclitaxel and doxorubicin under *in vitro* conditions. *Proteomics* **3:** (9) 1725.
Nagata T, Takahashia Y, Ishii Y, Asai S, Nishida Y, Murata A, Koshinaga T, Fukuzawa M, Hamazaki M, Asami K *et al*. 2003. Transcriptional profiling in hepatoblastomas using high-density oligonucleotide DNA array. *Cancer Genet Cytogenet* **145:** (2) 152.
Neville P, Tan PY, Mann G, Wolfinger R. 2003. Generalizable mass spectrometry mining used to identify disease state biomarkers from blood serum. *Proteomics* **3:** (9) 1710.
Nishizuka S, Chen ST, Gwadry FG, Alexander J, Major SM, Scherf U, Reinhold WC, Waltham M, Charboneau L, Young L *et al*. 2003. Diagnostic markers that distinguish colon and ovarian adenocarcinomas: Identification by genomic, proteomic, and tissue array profiling. *Cancer Res* **63:** (17) 5243.
Otsuka M, Arai M, Mori M, Kato M, Kato N, Yokosuka O, Ochiai T, Takiguchi M, Omata M, Seki N. 2003. Comparing gene expression profiles in human liver, gastric, and pancreatic tissues using full-length-enriched cDNA libraries. *Hepatol Res* **27:** (1) 76.
Park PC, Taylor MD, Mainprize TG, Becker LE, Ho M, Dura WT, Squire J, Rutka JT. 2003. Transcriptional profiling of medulloblastoma in children. *J Neurosurg* **99:** (3) 534.
Pedron T, Thibault C, Sansonetti PJ. 2003. The invasive phenotype of *Shigella flexneri* directs a distinct gene expression pattern in the human intestinal epithelial cell line Caco-2. *J Biol Chem* **278:** (36) 33878.
Peyrl A, Krapfenbauer K, Slavc I, Yang JW, Strobel T, Lubec G. 2003. Protein profiles of medulloblastoma cell lines DAOY and D283: Identification of tumor-related proteins and principles. *Proteomics* **3:** (9) 1781.
Porchet R, Probst A, Bouras C, Draberova E, Draber P, Riederer BM. 2003. Analysis of glial acidic fibrillary protein in the human entorhinal cortex during aging and in Alzheimer's disease. *Proteomics* **3:** (8) 1476.
Prieto D, Conrads TP, Scudiero DA, Veenstra TD, Issaq HJ. 2003. Profiling of secreted proteins from human ovarian cancer cell lines by surface-enhanced laser desorption/ionization time-of-flight mass spectrometry. *J Liq Chromatogr Relat Technol* **26:** (14) 2315.
Rehman KS, Yin S, Mayhew BA, Word RA, Rainey WE. 2003. Human myometrial adaptation to pregnancy: cDNA microarray gene expression profiling of myometrium from non-pregnant and pregnant women. *Mol Hum Reprod* **9:** (11) 681.
Robinson WH, Fontoura P, Lee BJ, De Vegvar HEN, Tom J, Pedotti R, DiGennaro CD, Mitchell DJ, Fong D, Ho PPK *et al*. 2003. Protein microarrays guide tolerizing DNA vaccine treatment of autoimmune encephalomyelitis. *Nat Biotechnol* **21:** (9) 1033.
Ryu JW, Kim HJ, Lee YS, Myong NH, Hwang CH, Lee GS, Yom HC. 2003. The proteomics approach to find biomarkers in gastric cancer. *J Korean Med Sci* **18:** (4) 505.
Sanchez JC, Converset V, Nolan A, Schmid G, Wang S, Heller M, Sennitt MV, Hochstrasser DF, Cawthorne MA. 2003. Effect of rosiglitazone on the differential expression of obesity and insulin resistance associated proteins in *lep/lep* mice. *Proteomics* **3:** (8) 1500.
Sotiriou C, Neo SY, McShane LM, Korn EL, Long PM, Jazaeri A, Martiat P, Fox SB, Harris AL, Liu ET. 2003. Breast cancer classification and prognosis based on gene expression profiles from a population-based study. *Proc Natl Acad Sci U S A* **100:** (18) 10393.
Stierum R, Gaspari M, Dommels Y, Ouatas T, Pluk H, Jespersen S, Vogels J, Verhoeckx K, Groten J, Van Ommen B. 2003. Proteome analysis reveals novel proteins associated with proliferation and differentiation of the colorectal cancer cell line Caco-2. *Biochim Biophys Acta* **1650:** (1-2) 73.
Takagaki K, Katsuma S, Horio T, Kaminishi Y, Hada Y, Tanaka T, Ohgi T, Yano J. 2003. cDNA microarray analysis of altered gene expression in Ara-C-treated leukemia cells. *Biochem Biophys Res Commun* **309:** (2) 351.
Unwin RD, Craven RA, Harnden P, Hanrahan S, Totty N, Knowles M, Eardley I, Selby PJ, Banks RE. 2003. Proteomic changes in renal cancer and co-ordinate demonstration of both the glycolytic and mitochondrial aspects of the Warburg effect. *Proteomics* **3:** (8) 1620.
Verjovski-Almeida S, DeMarco R, Martins EAL, Guimaraes PEM, Ojopi EPB, Paquola ACM, Piazza JP, Nishiyama MY, Kitajima JP, Adamson RE *et al*. 2003. Transcriptome analysis of the acoelomate human parasite *Schistosoma mansoni*. *Nat Genet* **35:** (2) 148.
Vuadens F, Benay C, Crettaz D, Gallot D, Sapin V, Schneider P, Bienvenut WV, Lemery D, Quadroni M, Dastugue B *et al*. 2003. Identification of biologic markers of the premature rupture of fetal membranes: Proteomic approach. *Proteomics* **3:** (8) 1521.
Wagner M, Naik D, Pothen A. 2003. Protocols for disease classification from mass spectrometry data. *Proteomics* **3:** (9) 1692.
Wang MZ, Howard B, Campa MJ, Patz EF, Fitzgerald MC. 2003. Analysis of human serum proteins by liquid phase isoelectric focusing and matrix-assisted laser desorption/ionization-mass spectrometry. *Proteomics* **3:** (9) 1661.
Xiao XY, Wei XP, He DC. 2003. Proteomic approaches to biomarker discovery in lung cancers by SELDI technology. *Sci China C Life Sci* **46:** (5) 531.
Ying H, Suzuki H, Furumoto H, Walker R, Meltzer P, Willingham MC, Cheng SY. 2003. Alterations in genomic profiles during tumor progression in a mouse model of follicular thyroid carcinoma. *Carcinogenesis* **24:** (9) 1467.
Yuan XL, Desiderio DM. 2003. Proteomics analysis of phosphotyrosyl-proteins in human lumbar cerebrospinal fluid. *J Proteome Res* **2:** (5) 476.
Yuza Y, Agawa M, Matsuzaki M, Yamada H, Urashima M. 2003. Gene and protein expression profiling differentiation of neuroblastoma cells triggered by 13-*cis* retinoic acid. *J Pediatr Hematol Oncol* **25:** (9) 715.
Zheng PP, Luider TM, Pieters R, Avezaat CJJ, Van den Bent MJ, Smitt PAES, Kros JM. 2003. Identification of tumor-related proteins by proteomic analysis of cerebrospinal fluid from patients with primary brain tumors. *J Neuropathol Exp Neurol* **62:** (8) 855.
Zhou Y, Luoh SM, Zhang Y, Watanabe C, Wu TD, Ostland M, Wood WI, Zhang ZM. 2003. Genome-wide identification of chromosomal regions of increased tumor expression by transcriptome analysis. *Cancer Res* **63:** (18) 5781.
Zhu HT, Yu CY, Zhang HP. 2003. Tree-based disease classification using protein data. *Proteomics* **3:** (9) 1673.

### 8 EST, cDNA and other clone resources

Chen SY, Jin WZ, Wang MY, Zhang F, Zhou J, Jia OJ, Wu YR, Liu FY, Wu P. 2003. Distribution and characterization of over 1000 T-DNA tags in rice genome. *Plant J* **36:** (1) 105.
Chen T, Reith ME, Ross NW, Macrae TH. 2003. Expressed sequence tag (EST)-based characterization of gene regulation in *Artemia larvae*. *Invertebr Reprod Dev* **44:** (1) 33.
De los Reyes BG, Morsy M, Gibbons J, Varma TSN, Antoine W, McGrath JM, Halgren R, Redus M. 2003. A snapshot of the low temperature stress transcriptome of developing rice seedlings (*Oryza sativa* L) *via* ESTs from subtracted cDNA library. *Theor Appl Genet* **107:** (6) 1071.
Gissi C, Pesole G. 2003. Transcript mapping and genome annotation of ascidian mtDNA using EST data. *Genome Res* **13:** (9) 2203.
Gong SC, Zheng C, Doughty ML, Losos K, Didkovsky N, Schambra

Copyright © 2004 John Wiley & Sons, Ltd.

UB, Nowak NJ, Joyner A, Leblanc G, Hatten ME *et al*. 2003. A gene expression atlas of the central nervous system based on bacterial artificial chromosomes. *Nature* **425:** (6961) 917.
Itoh T, Takasuga A, Watanabe T, Sugimoto Y. 2003. Mapping of 1400 expressed sequence tags in the bovine genome using a somatic cell hybrid panel. *Anim Genet* **34:** (5) 362.
Merino EF, Fernandez-Becerra C, Madeira AMBN, Machado AL, Durham A, Gruber A, Hall N, Del Portillo HA. 2003. Pilot survey of expressed sequence tags (ESTs) from the asexual blood stages of *Plasmodium vivax* in human patients. *Malaria J* **2:** 21.
Sacadura NT, Saville BJ. 2003. Gene expression and EST analyses of *Ustilago maydis* germinating teliospores. *Fungal Genet Biol* **40:** (1) 47.
Sawbridge T, Ong EK, Binnion C, Emmerling M, Meath K, Nunan K, O'Neill M, O'Toole F, Simmonds J, Wearne K *et al*. 2003. Generation and analysis of expressed sequence tags in white clover (*Trifolium repens* L). *Plant Sci* **165:** (5) 1077.
Sawbridge T, Ong EK, Binnion C, Emmerling M, McInnes R, Meath K, Nguyen N, Nunan K, O'Neill M, O'Toole F, et al. 2003. Generation and analysis of expressed sequence tags in perennial ryegrass (*Lolium perenne* L). *Plant Sci* **165:** (5) 1089.
Zhu W, Schlueter SD, Brendel V. 2003. Refined annotation of the *Arabidopsis* genome by complete expressed sequence tag mapping. *Plant Physiol* **132:** (2) 469.

### 9 Functional genomics

Amundson SA, Bittner M, Fornace AJ. 2003. Functional genomics as a window on radiation stress signaling. *Oncogene* **22:** (37) 5828.
Borner GHH, Lilley KS, Stevens TJ, Dupree P. 2003. Identification of glycosylphosphatidylinositol-anchored proteins in *Arabidopsis*. A proteomic and genomic analysis. *Plant Physiol* **132:** (2) 568.
Chanda SK, White S, Orth AP, Reisdorph R, Miraglia L, Thomas RS, DeJesus P, Mason DE, Huang QH, Vega R *et al*. 2003. Genome-scale functional profiling of the mammalian AP-1 signaling pathway. *Proc Natl Acad Sci U S A* **100:** (21) 12153.
Cuppen E, Van der Linden AM, Jansen G, Plasterk RHA. 2003. Proteins interacting with *Caenorhabditis elegans* G$\alpha$ subunits. *Comp Funct Genom* **4:** (5) 479.
Denef VJ, Park J, Rodrigues JLM, Tsoi TV, Hashsham SA, Tiedje JM. 2003. Validation of a more sensitive method for using spotted oligonucleotide DNA microarrays for functional genomics studies on bacterial communities. *Environ Microbiol* **5:** (10) 933.
Dorn G, Hall J, Husken D, Lange J, Martin P, Natt F, Wishart WL, Weiler J. 2003. Role for oligonucleotide-based RNA-knock down technologies in functional genomics. *Nucleosides Nucleotides* **22:** (5-8) 641.
Huang ME, Rio AG, Nicolas A, Kolodner RD. 2003. A genomewide screen in *Saccharomyces cerevisiae* for genes that suppress the accumulation of mutations. *Proc Natl Acad Sci U S A* **100:** (20) 11529.
Mi HY, Vandergriff J, Campbell M, Narechania A, Majoros W, Lewis S, Thomas PD, Ashburner M. 2003. Assessment of genome-wide protein function classification for *Drosophila melanogaster*. *Genome Res* **13:** (9) 2118.
Zhang YQ, Ren SX, Li HL, Wang YX, Fu G, Yang J, Qin ZQ, Miao YG, Wang WY, Chen RS *et al*. 2003. Genome-based analysis of virulence genes in a non-biofilm-forming *Staphylococcus epidermidis* strain (ATCC 12228). *Mol Microbiol* **49:** (6) 1577.

### I0 Transcriptomics

Anholt RRH, Dilda CL, Chang S, Fanara JJ, Kulkarni NH, Ganguly I, Rollmann SM, Kamdar KP, Mackay TFC. 2003. The genetic architecture of odor-guided behavior of *Drosophila*: Epistasis and the transcriptome. *Nat Genet* **35:** (2) 180.
Bailey MJ, Beremand PD, Hammer R, Bell-Pedersen D, Thomas TL, Cassone VM. 2003. Transcriptional profiling of the chick pineal gland, a photoreceptive circadian oscillator and pacemaker. *Mol Endocrinol* **17:** (10) 2084.
Becerra M, Lombardia LJ, Gonzalez-Siso MI, Rodriguez-Belmonte E, Hauser NC, Cerdan ME. 2003. Genome-wide analysis of the yeast tran scriptome upon heat and cold shock. *Comp Funct Genom* **4:** (4) 366.
Birch PRJ, Avrova AO, Armstrong M, Venter E, Taleb N, Gilroy EM, Phillips MS, Whisson SC. 2003. The potato: *Phytophthora infestans* interaction transcriptome. *Can J Plant Pathol* **25:** (3) 226.
Bucca G, Brassington AME, Hotchkiss G, Mersinias V, Smith CP. 2003. Negative feedback regulation of *dnaK*, *clpB* and *lon* expression by the DnaK chaperone machine in *Streptomyces coelicolor*, identified by transcriptome and *in vivo* DnaK-depletion analysis. *Mol Microbiol* **50:** (1) 153.
Chen IP, Haehnel U, Altschmied L, Schubert I, Puchta H. 2003. The transcriptional response of *Arabidopsis* to genotoxic stress: A high-density colony array study (HDCA). *Plant J* **35:** (6) 771.
De Parseval N, Lazar V, Casella JF, Benit L, Heidmann T. 2003. Survey of human genes of retroviral origin: Identification and transcriptome of the genes with coding capacity for complete envelope proteins. *J Virol* **77:** (19) 10414.
Espanhol AR, Macedo C, Junta CM, Cardoso RS, Victorero G, Loriod B, Nguyen C, Jordan B, Passos GAS. 2003. Gene expression profiling during thymus ontogeny and its association with TCRV$\beta$8.1-D$\beta$2.1 rearrangements of inbred mouse strains. *Mol Cell Biochem* **252:** (1-2) 223.
Halitschke R, Gase K, Hui DQ, Schmidt DD, Baldwin IT. 2003. Molecular interactions between the specialist herbivore *Manduca sexta* (Lepidoptera, Sphingidae) and its natural host *Nicotiana attenuata*. VI. Microarray analysis reveals that most herbivore-specific transcriptional changes are mediated by fatty acid-amino acid conjugates. *Plant Physiol* **131:** (4) 1894.
Honys D, Twell D. 2003. Comparative analysis of the *Arabidopsis* pollen transcriptome. *Plant Physiol* **132:** (2) 640.
Hui DQ, Iqbal J, Lehmann K, Gase K, Saluz HP, Baldwin IT. 2003. Molecular interactions between the specialist herbivore *Manduca* sexta (Lepidoptera, Sphingidae) and its natural host *Nicotiana attenuata*: V. Microarray analysis and further characterization of large-scale changes in herbivore-induced mRNAs. *Plant Physiol* **131:** (4) 1877.
Lee JH, Lee DE, Lee BU, Kim HS. 2003. Global analyses of transcriptomes and proteomes of a parent strain and an L-threonine overproducing mutant strain. *J Bacteriol* **185:** (18) 5442.
Lee JY, Lee DH. 2003. Use of serial analysis of gene expression technology to reveal changes in gene expression in *Arabidopsis* pollen undergoing cold stress. *Plant Physiol* **132:** (2) 517.
Savaldi-Goldstein S, Aviv D, Davydov O, Fluhr R. 2003. Alternative splicing modulation by a LAMMER kinase impinges on developmental and transcriptome expression. *Plant Cell* **15:** (4) 926.
Steil L, Hoffmann T, Budde I, Volker U, Bremer E. 2003. Genome-wide transcriptional profiling analysis of adaptation of *Bacillus subtilis* to high salinity. *J Bacteriol* **185:** (21) 6358.
Utaida S, Dunman PM, Macapagal D, Murphy E, Projan SJ, Singh VK, Jayaswal RK, Wilkinson BJ. 2003. Genome-wide transcriptional profiling of the response of *Staphylococcus aureus* to cell-wall-active antibiotics reveals a cell-wall-stress stimulon. *Microbiology* **149:** (10) 2719.
Van Wees SCM, Chang HS, Zhu T, Glazebrook J. 2003. Characterization of the early response of *Arabidopsis* to *Alternaria brassicicola* infection using expression profiling. *Plant Physiol* **132:** (2) 606.
Vilaine F, Palauqui JC, Amselem J, Kusiak C, Lemoine R, Dinant S. 2003. Towards deciphering phloem: A transcriptome analysis of the phloem of *Apium graveolens*. *Plant J* **36:** (1) 67.
Wang RC, Okamoto M, Xing XJ, Crawford NM. 2003. Microarray analysis of the nitrate response in *Arabidopsis* roots and shoots reveals over 1,000 rapidly responding genes and new linkages to glucose, trehalose-6-phosphate, iron, and sulfate metabolism. *Plant Physiol* **132:** (2) 556.
Wasaki J, Yonetani R, Kuroda S, Shinano T, Yazaki J, Fujii F, Shimbo K, Yamamoto K, Sakata K, Sasaki T *et al*. 2003. Transcriptomic analysis of metabolic changes by phosphorus stress in rice plant roots. *Plant Cell Environ* **26:** (9) 1515.
Wendisch VF. 2003. Genome-wide expression analysis in *Corynebacterium glutamicum* using DNA microarrays. *J Biotechnol* **104:** (1-3) 273.
Wride MA, Mansergh FC, Adams S, Everitt R, Minnema SE, Rancourt DE, Evans MJ. 2003. Expression profiling and gene discovery in the mouse lens. *Mol Vis* **9:** (50) 360.
Wu CF, Valdes JJ, Bentley WE, Sekowski JW. 2003. DNA microarray for discrimination between pathogenic O157:H7 EDL933 and non-pathogenic *Escherichia coli* strains. *Biosens Bioelectron* **19:** (1) 1.

Copyright © 2004 John Wiley & Sons, Ltd. 

Wu P, Ma LG, Hou XL, Wang MY, Wu YR, Liu FY, Deng XW. 2003. Phosphate starvation triggers distinct alterations of genome expression in *Arabidopsis* roots and leaves. *Plant Physiol* **132:** (3) 1260.

Yamada K, Lim J, Dale JM, Chen H, Shinn P, Palm CJ, Southwick AM, Wu H, Kim C, Nguyen M *et al*. 2003. Empirical analysis of transcriptional activity in the *Arabidopsis* genome. *Science* **302:** (5646) 842.

Yang JM, Park S, Kamdem DP, Keathley DE, Retzel E, Paule C, Kapur V, Han KH. 2003. Novel gene expression profiles define the metabolic and physiological processes characteristic of wood and its extractive formation in a hardwood tree species, *Robinia pseudoacacia*. *Plant Mol Biol* **52:** (5) 935.

Yoon BI, Li GX, Kitada K, Kawasaki Y, Igarashi K, Kodama Y, Inoue T, Kobayashi K, Kanno J, Kim DY *et al*. 2003. Mechanisms of benzene-induced hematotoxicity and leukemogenicity: cDNA microarray analyses using mouse bone marrow tissue. *Environ Health Perspect* **111:** (11) 1411.

### II Proteomics

Bendt AK, Burkovski A, Schaffer S, Bott M, Farwick M, Hermann T. 2003. Towards a phosphoproteome map of *Corynebacterium glutamicum*. *Proteomics* **3:** (8) 1637.

Binz PA, Abdi F, Affolter M, Allard L, Barblan J, Bhardwaj S, Bienvenut WV, Bulet P, Burgess J, Carrette O *et al*. 2003. Proteomics application exercise of the Swiss Proteomics Society: Report of the SPS'02 session. *Proteomics* **3:** (8) 1562.

Braun P, LaBaer J. 2003. High throughput protein production for functional proteomics. *Trends Biotechnol* **21:** (9) 383.

Brock JWC, Hinton DJS, Cotham WE, Metz TO, Thorpe SR, Baynes JW, Ames JM. 2003. Proteomic analysis of the site specificity of glycation and carboxymethylation of ribonuclease. *J Proteome Res* **2:** (5) 506.

Bromley E, Leeds N, Clark J, McGregor E, Ward M, Dunn MJ, Tomley F. 2003. Defining the protein repertoire of microneme organelles in the apicomplexan parasite secretor or *Eimeria tenefla*. *Proteomics* **3:** (8) 1553.

Che FY, Eipper BA, Mains RE, Fricker LD. 2003. Quantitative peptidomics of pituitary glands from mice deficient in copper transport. *Cell Mol Biol (Noisy-le-Grand)* **49:** (5) 713.

Chemale G, Van Rossum AJ, Jefferies JR, Barrett J, Brophy PM, Ferreira HB, Zaha A. 2003. Proteomic analysis of the larval stage of the parasite *Echinococcus granulosus*: Causative agent of cystic hydatid disease. *Proteomics* **3:** (8) 1633.

Colinge J, Magnin J, Dessingy T, Giron M, Masselot A. 2003. Improved peptide charge state assignment. *Proteomics* **3:** (8) 1434.

Damoc E, Youhnovski N, Crettaz D, Tissot JD, Przybylski M. 2003. High resolution proteome analysis of cryoglobulins using Fourier transform-ion cyclotron resonance mass spectrometry. *Proteomics* **3:** (8) 1425.

Duzhak T, Emerson MR, Chakrabarty A, Alterman MA, Levine SM. 2003. Analysis of protein induction in the CNS of SJL mice with experimental allergic encephalomyelitis by proteomic screening and immunohistochemistry. *Cell Mol Biol (Noisy-le-Grand)* **49:** (5) 723.

He XY, Zhuang YH, Zhang XG, Li GL. 2003. Comparative proteome analysis of culture supernatant proteins of *Mycobacterium tuberculosis* H37Rv and H37Ra. *Microbes Infect* **5:** (10) 851.

Heazlewood JL, Howell KA, Whelan J, Millar AH. 2003. Towards an analysis of the rice mitochondrial proteome. *Plant Physiol* **132:** (1) 230.

Hestvik ALK, Hmama Z, Av-Gay Y. 2003. Kinome analysis of host response to mycobacterial infection: A novel technique in proteomics. *Infect Immun* **71:** (10) 5514.

Kerk D, Bulgrien J, Smith DW, Gribskov M. 2003. *Arabidopsis* proteins containing similarity to the universal stress protein domain of bacteria. *Plant Physiol* **131:** (3) 1209.

Krayl M, Benndorf D, Loffhagen N, Babel W. 2003. Use of proteomics and physiological characteristics to elucidate ecotoxic effects of methyl tert-butyl ether in *Pseudomonas putida* KT2440. *Proteomics* **3:** (8) 1544.

Liao XA, Ying TY, Wang HL, Wang J, Shi ZX, Feng EL, Wei KH, Wang YL, Zhang XM, Huang LY *et al*. 2003. A two-dimensional proteome map of *Shigella flexneri*. *Electrophoresis* **24:** (16) 2864.

Madi A, Mikkat S, Ringel B, Ulbrich M, Thiesen HJ, Glocker MO. 2003. Mass spectrometric proteome analysis for profiling temperature-dependent changes of protein expression in wild type *Caenorhabditis elegans*. *Proteomics* **3:** (8) 1526.

Mamone G, Caira S, Garro G, Nicolai A, Ferranti P, Picariello G, Malorni A, Chianese L, Addeo F. 2003. Casein phosphoproteome: Identification of phosphoproteins by combined mass spectrometry and two-dimensional gel electrophoresis. *Electrophoresis* **24:** (16) 2824.

Maruyama N, Fukuda T, Saka S, Inui N, Kotoh J, Miyagawa M, Hayashi M, Sawada M, Moriyama T, Utsumi S. 2003. Molecular and structural analysis of electrophoretic variants of soybean seed storage proteins. *Phytochemistry* **64:** (3) 701.

Nawarak J, Sinchaikul S, Wu CY, Liau MY, Phutrakul S, Chen ST. 2003. Proteomics of snake venoms from Elapidae and Viperidae families by multidimensional chromatographic methods. *Electrophoresis* **24:** (16) 2838.

Quach TTT, Li N, Richards DP, Zheng J, Keller BO, Li L. 2003. Development and applications of in-gel CNBr/tryptic digestion combined with mass spectrometry for the analysis of membrane proteins. *J Proteome Res* **2:** (5) 543.

Rexroth S, Tittingdorf JMWMZ, Krause F, Dencher NA, Seelert H. 2003. Thylakoid membrane at altered metabolic state: Challenging the forgotten realms of the proteome. *Electrophoresis* **24:** (16) 2814.

Reymond MA, Steinert R, Eder F, Lippert H. 2003. Ethical and regulatory issues arising from proteomic research and technology. *Proteomics* **3:** (8) 1387.

Rodriguez-Ortega MJ, Grosvik BE, Rodriguez-Ariza A, Goksoyr A, Lopez-Barea J. 2003. Changes in protein expression profiles in bivalve molluscs (*Chamaelea gallina*) exposed to four model environmental pollutants. *Proteomics* **3:** (8) 1535.

Scheffler NK, Falick AM, Hall SC, Ray WC, Post DM, Munson RS, Gibson BW. 2003. Proteome of *Haemophilus ducreyi* by 2-D SDS-PAGE and mass spectrometry: Strain variation, virulence, and carbohydrate expression. *J Proteome Res* **2:** (5) 523.

Sebastiano R, Citterio A, Lapadula M, Righetti PG. 2003. A new deuterated alkylating agent for quantitative proteomics. *Rapid Commun Mass Spectrom* **17:** (21) 2380.

Tien CC, Chen JB, Wang CC, Lee WC. 2003. Preliminary proteome analysis of rabbit serum with hepatic failure. *Enzyme Microb Technol* **33:** (4) 488.

Umar A, Ooms MP, Luider TM, Grootegoed JA, Brinkmann AO. 2003. Proteomic profiling of epididymis and vas deferens: Identification of proteins regulated during rat genital tract development. *Endocrinology* **144:** (10) 4637.

Van Lis R, Atteia A, Mendoza-Hernandez G, Gonzalez-Halphen D. 2003. Identification of novel mitochondrial protein components of *Chlamydomonas reinhardtii*. A proteomic approach. *Plant Physiol* **132:** (1) 318.

Walcher W, Franze T, Weller MG, Poschl U, Huber CG. 2003. Liquid- and gas-phase nitration of bovine serum albumin studied by LC-MS and LC-MS/MS using monolithic columns. *J Proteome Res* **2:** (5) 534.

Watson BS, Asirvatham VS, Wang LJ, Sumner LW. 2003. Mapping the proteome of barrel medic (*Medicago truncatula*). *Plant Physiol* **131:** (3) 1104.

Weiler T, Sauder P, Cheng K, Ens W, Standing K, Wilkins JA. 2003. A proteomics-based approach for monoclonal antibody characterization. *Anal Biochem* **321:** (2) 217.

Wienkoop S, Saalbach G. 2003. Proteome analysis. Novel proteins identified at the peribacteroid membrane from *Lotus japonicus* root nodules. *Plant Physiol* **131:** (3) 1080.

Zhang W, Zhou G, Zhao YX, White MA, Zhao YM. 2003. Affinity enrichment of plasma membrane for proteomics analysis. *Electrophoresis* **24:** (16) 2855.

Zhong JP, Haynes PA, Zhang SP, Yang XP, Andon NL, Eckert D, Yates JR, Wang X, Budworth P. 2003. Development of a system for the study of protein-protein interactions in planta: Characterization of a TATA-box binding protein complex in *Oryza sativa*. *J Proteome Res* **2:** (5) 514.

### I2 Protein structural genomics

Joshi RR, Jyothi S. 2003. *Ab-initio* prediction and reliability of protein structural genomics by PROPAINOR algorithm. *Comput Biol Chem* **27:** (3) 241.

Copyright © 2004 John Wiley & Sons, Ltd. 

### I3 Metabolomics

Beisson F, Koo AJK, Ruuska S, Schwender J, Pollard M, Thelen JJ, Paddock T, Salas JJ, Savage L, Milcamps A *et al*. 2003. *Arabidopsis* genes involved in acyl lipid metabolism. A 2003 census of the candidates, a study of the distribution of expressed sequence tags in organs, and a web-based database. *Plant Physiol* **132:** (2) 681.
Hattori M, Okuno Y, Goto S, Kanehisa M. 2003. Development of a chemical structure comparison method for integrated analysis of chemical and genomic information in the metabolic pathways. *J Am Chem Soc* **125:** (39) 11853.
Kaderbhai NN, Broadhurst DI, Ellis DI, Goodacre R, Kell DB. 2003. Functional genomics *via* metabolic footprinting: Monitoring metabolite secretion by *Escherichia coli* tryptophan metabolism mutants using FT-IR and direct injection electrospray mass spectrometry. *Comp Funct Genom* **4:** (4) 376.
Maruyama-Nakashita A, Inoue E, Watanabe-Takahashi A, Yarnaya T, Takahashi H. 2003. Transcriptome profiling of sulfur-responsive genes in *Arabidopsis* reveals global effects of sulfur nutrition on multiple metabolic pathways. *Plant Physiol* **132:** (2) 597.
Ruckert C, Puhler A, Kalinowski J. 2003. Genome-wide analysis of the L-methionine biosynthetic pathway in *Corynebacterium glutamicum* by targeted gene deletion and homologous complementation. *J Biotechnol* **104:** (1-3) 213.
Soga T, Ohashi Y, Ueno Y, Naraoka H, Tomita M, Nishioka T. 2003. Quantitative metabolome analysis using capillary electrophoresis mass-spectrometry. *J Proteome Res* **2:** (5) 488.
Urbanczyk-Wochniak E, Luedemann A, Kopka J, Selbig J, Roessner-Tunali U, Willmitzer L, Fernie AR. 2003. Parallel analysis of transcript and metabolic profiles: A new approach in systems biology. *EMBO Rep* **4:** (10) 989.

### I4 Genomic approaches to development

Bente M, Harder S, Wiesgigl M, Heukeshoven J, Gelhaus C, Krause E, Clos J, Bruchhaus I. 2003. Developmentally induced changes of the proteome in the protozoan parasite *Leishmania donovani*. *Proteomics* **3:** (9) 1811.
Liu JY, Blaylock LA, Endre G, Cho J, Town CD, Van den Bosch KA, Harrison MJ. 2003. Transcript profiling coupled with spatial expression analyses reveals genes involved in distinct developmental stages of an arbuscular mycorrhizal symbiosis. *Plant Cell* **15:** (9) 2106.
Thibaud-Nissen FO, Shealy RT, Khanna A, Vodkin LO. 2003. Clustering of microarray data reveals transcript patterns associated with somatic embryogenesis in soybean. *Plant Physiol* **132:** (1) 118.

### I5 Technological advances

Aldridge BA, Lim SD, Baumann AK, Hosseini S, Buck W, Almekinder TL, Sun CQ, Petros JA. 2003. Automated sequencing of complete mitochondrial genomes from laser-capture microdissected samples. *Biotechniques* **35:** (3) 606.
Astorga-Wells J, Jornvall H, Bergman T. 2003. A microfluidic electrocapture device in sample preparation for protein analysis by MALDI mass spectrometry. *Anal Chem* **75:** (19) 5213.
Bertsch M, Kassner RJ. 2003. Selective staining of proteins with hydrophobic surface sites on a native electrophoretic gel. *J Proteome Res* **2:** (5) 469.
Bunai K, Nozaki M, Hamano M, Ogane S, Inoue T, Nemoto T, Nakanishi H, Yamane K. 2003. Proteomic analysis of acrylamide gel separated proteins immobilized on polyvinylidene difluoride membranes following proteolytic digestion in the presence of 80% acetonitrile. *Proteomics* **3:** (9) 1738.
Deshusses JMP, Burgess JA, Scherl A, Wenger Y, Walter N, Converset V, Paesano S, Corthals GL, Hochstrasser DF, Sanchez JC. 2003. Exploitation of specific properties of trifluoroethanol for extraction and separation of membrane proteins. *Proteomics* **3:** (8) 1418.
Evans EC, Horn T, Wagner MA, Eschenbrenner M, Mujer CV, Del Vecchio VG. 2003. Isolation protocol for two-dimensional-polyacrylamide gel electrophoresis analysis of *Haloferax volcanii* proteome. *Biotechniques* **35:** (3) 478.
Gentzel M, Kocher T, Ponnusamy S, Wilm M. 2003. Preprocessing of tandem mass spectrometric data to support automatic protein identification. *Proteomics* **3:** (8) 1597.
Guimil R, Beier M, Scheffler M, Rebscher H, Funk J, Wixmerten A, Baum M, Hermann C, Tahedl H, Moschel E *et al*. 2003. Geniom® technology: The benchtop array facility. *Nucleosides Nucleotides* **22:** (5-8) 1721.
Hunter TC, Washburn MP. 2003. Integration of chromatography and peptide mass modification for quantitative proteomics. *J Liq Chromatogr Relat Technol* **26:** (14) 2285.
Issaq HJ, Fox SD, Mahadevan M, Conrads TP, Veenstra TD. 2003. Effect of experimental parameters on the HPLC separation of peptides and proteins. *J Liq Chromatogr Relat Technol* **26:** (14) 2255.
McClure J, Wit E. 2003. Post-normalization quality assessment visualization of microarray data. *Comp Funct Genom* **4:** (5) 460.
Millea KM, Krull IS. 2003. Subproteomics in analytical chemistry: Chromatographic fractionation techniques in the characterization of proteins and peptides. *J Liq Chromatogr Relat Technol* **26:** (14) 2195.
Mohan D, Pasa-Tolic L, Masselon CD, Tolic N, Bogdanov B, Hixson KK, Smith RD, Lee CS. 2003. Integration of electrokinetic-based multidimensional separation/concentration platform with electrospray ionization-Fourier transform ion cyclotron resonance-mass spectrometry for proteome analysis of *Shewanella oneidensis*. *Anal Chem* **75:** (17) 4432.
Nakazono M, Qiu F, Borsuk LA, Schnable PS. 2003. Laser-capture microdissection, a tool for the global analysis of gene expression in specific plant cell types: Identification of genes expressed differentially in epidermal cells or vascular tissues of maize. *Plant Cell* **15:** (3) 583.
Nemeth-Cawley JF, Tangarone BS, Rouse JC. 2003. "Top Down" characterization is a complementary technique to peptide sequencing for identifying protein species in complex mixtures. *J Proteome Res* **2:** (5) 495.
Purohit PV, Rocke DM. 2003. Discriminant models for high-throughput proteomics mass spectrometer data. *Proteomics* **3:** (9) 1699.
Righetti PG, Castagna A, Herbert B, Reymond F, Rossier JS. 2003. Prefractionation techniques in proteome analysis. *Proteomics* **3:** (8) 1397.
Shaw MM, Riederer BM. 2003. Sample preparation for two-dimensional gel electrophoresis. *Proteomics* **3:** (8) 1408.
Slotta DJ, Heath LS, Ramakrishnan N, Helm R, Potts M. 2003. Clustering mass spectrometry data using order statistics. *Proteomics* **3:** (9) 1687.
Starita-Geribaldi M, Roux F, Garin J, Chevallier D, Fenichel P, Pointis G. 2003. Development of narrow immobilized pH gradients covering one pH unit for human seminal plasma proteomic analysis. *Proteomics* **3:** (8) 1611.
Tan PK, Downey TJ, Spitznagel EL, Xu P, Fu D, Dimitrov DS, Lempicki RA, Raaka BM, Cam MC. 2003. Evaluation of gene expression measurements from commercial microarray platforms. *Nucleic Acids Res* **31:** (19) 5676.
Washburn MP, Ulaszek RR, Yates JR. 2003. Reproducibility of quantitative proteomic analyses of complex biological mixtures by multidimensional protein identification technology. *Anal Chem* **75:** (19) 5054.
Zhong WW, Yeung ES. 2003. High-throughput analysis of total RNA expression profiles by capillary gel electrophoresis. *Anal Chem* **75:** (17) 4415.

### I6 Bioinformatics

Allen TE, Herrgard MJ, Liu MZ, Qiu Y, Glasner JD, Blattner FR, Palsson BO. 2003. Genome-scale analysis of the uses of the *Escherichia coli* genome: Model-driven analysis of heterogeneous data sets. *J Bacteriol* **185:** (21) 6392.
Autio R, Hautaniemi S, Kauraniemi P, Yli-Harja O, Astola J, Wolf M, Kallioniemi A. 2003. CGH-Plotter: MATLAB toolbox for CGH-data analysis. *Bioinformatics* **19:** (13) 1714.
Bagirov AM, Ferguson B, Ivkovic S, Saunders G, Yearwood J. 2003. New algorithms for multi-class cancer diagnosis using tumor gene expression signatures. *Bioinformatics* **19:** (14) 1800.
Bar-Joseph Z, Gerber G, Simon L, Gifford DK, Jaakkola TS, Jaakkola TS. 2003. Comparing the continuous representation of time-series expression profiles to identify differentially expressed genes. *Proc Natl Acad Sci U S A* **100:** (18) 10146.

Copyright © 2004 John Wiley & Sons, Ltd. 

Barker JA, Thornton JM. 2003. An algorithm for constraint-based structural template matching: Application to 3D templates with statistical analysis. *Bioinformatics* **19:** (13) 1644.
Blick RJ, Revel AT, Hansen EJ. 2003. FindGDPs: Identification of primers for labeling microbial transcriptomes for DNA microarray analysis. *Bioinformatics* **19:** (13) 1718.
Carver T, Bleasby A. 2003. The design of Jemboss: A graphical user interface to EMBOSS. *Bioinformatics* **19:** (14) 1837.
Cole SW, Galic Z, Zack JA. 2003. Controlling false-negative errors in microarray differential expression analysis: A PRIM approach. *Bioinformatics* **19:** (14) 1808.
Colinge J, Masselot A, Giron M, Dessingy T, Magnin J. 2003. OLAV: Towards high-throughput tandem mass spectrometry data identification. *Proteomics* **3:** (8) 1454.
Creevey CJ, McInerney JO. 2003. CRANN: Detecting adaptive evolution in protein-coding DNA sequences. *Bioinformatics* **19:** (13) 1726.
Date SV, Marcotte EM. 2003. Discovery of uncharacterized cellular systems by genome-wide analysis of functional linkages. *Nat Biotechnol* **21:** (9) 1055.
Dhillon IS, Marcotte EM, Roshan U. 2003. Diametrical clustering for identifying anti-correlated gene clusters. *Bioinformatics* **19:** (13) 1612.
Di Bernardo D, Down T, Hubbard T. 2003. ddbRNA: Detection of conserved secondary structures in multiple alignments. *Bioinformatics* **19:** (13) 1606.
Firneisz G, Zehavi I, Vermes C, Hanyecz A, Frieman JA, Glant TT. 2003. Identification and quantification of disease-related gene clusters. *Bioinformatics* **19:** (14) 1781.
Gabdoulline RR, Hoffmann R, Leitner F, Wade RC. 2003. ProSAT: functional annotation of protein 3D structures. *Bioinformatics* **19:** (13) 1723.
Galinsky VL. 2003. Automatic registration of microarray images. I. Rectangular grid. *Bioinformatics* **19:** (14) 1824.
Galinsky VL. 2003. Automatic registration of microarray images. II. Hexagonal grid. *Bioinformatics* **19:** (14) 1832.
Geller SC, Gregg JP, Hagerman P, Rocke DM. 2003. Tranformation and normalization of oligonucleotide microarray data. *Bioinformatics* **19:** (14) 1817.
Guo X, Qi HL, Verfaillie CM, Pan W. 2003. Statistical significance analysis of longitudinal gene expression data. *Bioinformatics* **19:** (13) 1628.
Harrison A, Pearl F, Silitoe I, Slidel T, Mott R, Thornton J, Orengo C. 2003. Recognizing the fold of a protein structure. *Bioinformatics* **19:** (14) 1748.
Hering JA, Innocent PR, Haris PI. 2003. Neuro-fuzzy structural classification of proteins for improved protein secondary structure prediction. *Proteomics* **3:** (8) 1464.
Herron-Olson L, Freeman J, Zhang Q, Retzel EF, Kapur V. 2003. MGView: An alignment and visualization tool to enhance gap closure of microbial genomes: Online art. no. e106. *Nucleic Acids Res* **31:** (17) e106.
Huang XQ, Wang JM, Aluru S, Yang SP, Hillier L. 2003. PCAP: A whole-genome assembly program. *Genome Res* **13:** (9) 2164.
Huntley D, Hummerich H, Smedley D, Kittivoravitkul S, McCarthy M, Little P, Sergot M. 2003. GANESH: Software for customized annotation of genome regions. *Genome Res* **13:** (9) 2195.
Jansen R, Yu HY, Greenbaum D, Kluger Y, Krogan NJ, Chung SB, Emili A, Snyder M, Greenblatt JF, Gerstein M. 2003. A Bayesian networks approach for predicting protein-protein interactions from genomic data. *Science* **302:** (5644) 449.
Jones A, Wastling J, Hunt E. 2003. Proposal for a standard representation of two-dimensional gel electrophoresis data. *Comp Funct Genom* **4:** (5) 492.
Kreil DP, Ouzounis CA. 2003. Comparison of sequence masking algorithms and the detection of biased protein sequence regions. *Bioinformatics* **19:** (13) 1672.
Laws RJ, Bergemann TL, Quiaoit F, Zhao LP. 2003. SignalViewer: Analyzing microarray images. *Bioinformatics* **19:** (13) 1716.
Lee KR, Lin XW, Park DC, Eslava S. 2003. Megavariate data analysis of mass spectrometric proteomics data using latent variable projection method. *Proteomics* **3:** (9) 1680.
Leinonen R, Nardone F, Oyewole O, Redaschi N, Stoehr P. 2003. The EMBL sequence version archive. *Bioinformatics* **19:** (14) 1861.
Li L, Stoeckert CJ, Roos DS. 2003. OrthoMCL: Identification of ortholog groups for eukaryotic genomes. *Genome Res* **13:** (9) 2178.
Li SY, Cutler G, Liu JJJ, Hoey T, Chen LB, Schultz PG, Liao JY, Ling XFB. 2003. A comparative analysis of HGSC and Celera human genome assemblies and gene sets. *Bioinformatics* **19:** (13) 1597.
Mao CH, Cushman JC, May GD, Weller JW. 2003. ESTAP - an automated system for the analysis of EST data. *Bioinformatics* **19:** (13) 1720.
Marongiu A, Palazzari P, Rosato V. 2003. Designing hardware for protein sequence analysis. *Bioinformatics* **19:** (14) 1739.
McShan DC, Rao S, Shah I. 2003. PathMiner: Predicting metabolic pathways by heuristic search. *Bioinformatics* **19:** (13) 1692.
Medjahed D, Luke BT, Tontesh TS, Smythers GW, Munroe DJ, Lemkin PF. 2003. Tissue Molecular Anatomy Project (TMAP): An expression database for comparative cancer proteomics. *Proteomics* **3:** (8) 1445.
Mitchell AL, Reich JR, Attwood TK. 2003. PRECIS: Protein reports engineered from concise information in SWISS-PROT. *Bioinformatics* **19:** (13) 1664.
Mooney SD, Altman RB. 2003. MutDB: Annotating human variation with functionally relevant data. *Bioinformatics* **19:** (14) 1858.
Mostaguir K, Hoogland C, Binz PA, Appel RD. 2003. The Make 2D-DB II package: Conversion of federated two-dimensional gel electrophoresis databases into a relational format and interconnection of distributed databases. *Proteomics* **3:** (8) 1441.
Parisi V, De Fonzo V, Aluffi-Pentini F. 2003. STRING: finding tandem repeats in DNA sequences. *Bioinformatics* **19:** (14) 1733.
Park KJ, Kanehisa M. 2003. Prediction of protein subcellular locations by support vector machines using compositions of amino acids and amino acid pairs. *Bioinformatics* **19:** (13) 1656.
Pavlidis P, Li QH, Noble WS. 2003. The effect of replication on gene expression microarray experiments. *Bioinformatics* **19:** (13) 1620.
Pichon C, Felden B. 2003. Intergenic sequence inspector: Searching and identifying bacterial RNAs. *Bioinformatics* **19:** (13) 1707.
Prasad JC, Comeau SR, Vajda S, Camacho CJ. 2003. Consensus alignment for reliable framework prediction in homology modeling. *Bioinformatics* **19:** (13) 1682.
Shah NH, King DC, Shah PN, Fedoroff NV. 2003. A tool-kit for cDNA microarray and promoter analysis. *Bioinformatics* **19:** (14) 1846.
Sharan R, Maron-Katz A, Shamir R. 2003. CLICK and EXPANDER: A system for clustering and visualizing gene expression data. *Bioinformatics* **19:** (14) 1787.
Takahashi K, Ishikawa N, Sadamoto Y, Sasamoto H, Ohta S, Shiozawa A, Miyoshi F, Naito Y, Nakayama Y, Tomita M. 2003. E-Cell 2: Multi-platform E-Cell simulation system. *Bioinformatics* **19:** (13) 1727.
Tatay JW, Feng X, Sobczak N, Jiang H, Chen CF, Kirova R, Struble C, Wang NJ, Tonellato PJ. 2003. Multiple approaches to data-mining of proteomic data based on statistical and pattern classification methods. *Proteomics* **3:** (9) 1704.
Thomas PD, Campbell MJ, Kejariwal A, Mi HY, Karlak B, Daverman R, Diemer K, Muruganujan A, Narechania A. 2003. PANTHER: A library of protein families and subfamilies indexed by function. *Genome Res* **13:** (9) 2129.
Tjandra D, Wong S, Shen WM, Pulliam B, Yu E, Esserman L. 2003. An XML message broker framework for exchange and integration of microarray data. *Bioinformatics* **19:** (14) 1844.
Van Nimwegen E. 2003. Scaling laws in the functional content of genomes. *Trends Genet* **19:** (9) 479.
Vinayagam A, Shi J, Pugalenthi G, Meenakshi B, Blundell TL, Sowdhamini R. 2003. DDBASE2.0: Updated domain database with improved identification of structural domains. *Bioinformatics* **19:** (14) 1760.
Wall DP, Fraser HB, Hirsh AE. 2003. Detecting putative orthologs. *Bioinformatics* **19:** (13) 1710.
Wang J, Kraemer E. 2003. GFPE: Gene-finding program evaluation. *Bioinformatics* **19:** (13) 1712.
Ward JJ, McGuffin LJ, Buxton BF, Jones DT. 2003. Secondary structure prediction with support vector machines. *Bioinformatics* **19:** (13) 1650.
Wu BL, Abbott T, Fishman D, McMurray W, Mor G, Stone K, Ward D, Williams K, Zhao HY. 2003. Comparison of statistical methods for classification of ovarian cancer using mass spectrometry data. *Bioinformatics* **19:** (13) 1636.
Xu D, Olman V, Wang L, Xu Y. 2003. EXCAVATOR: A computer program for efficiently mining gene expression data. *Nucleic Acids Res* **31:** (19) 5582.

Copyright © 2004 John Wiley & Sons, Ltd. 
